# Supplementary figures and images for: Assessment of physical status and analysis of lipidomic and metabolomic alterations in patients with Post-COVID-19 condition
Source: PLoS One. 2026 Mar 3;21(3):e0341192. doi: 10.1371/journal.pone.0341192 (PMC12956072; doi:10.1371/journal.pone.0341192)

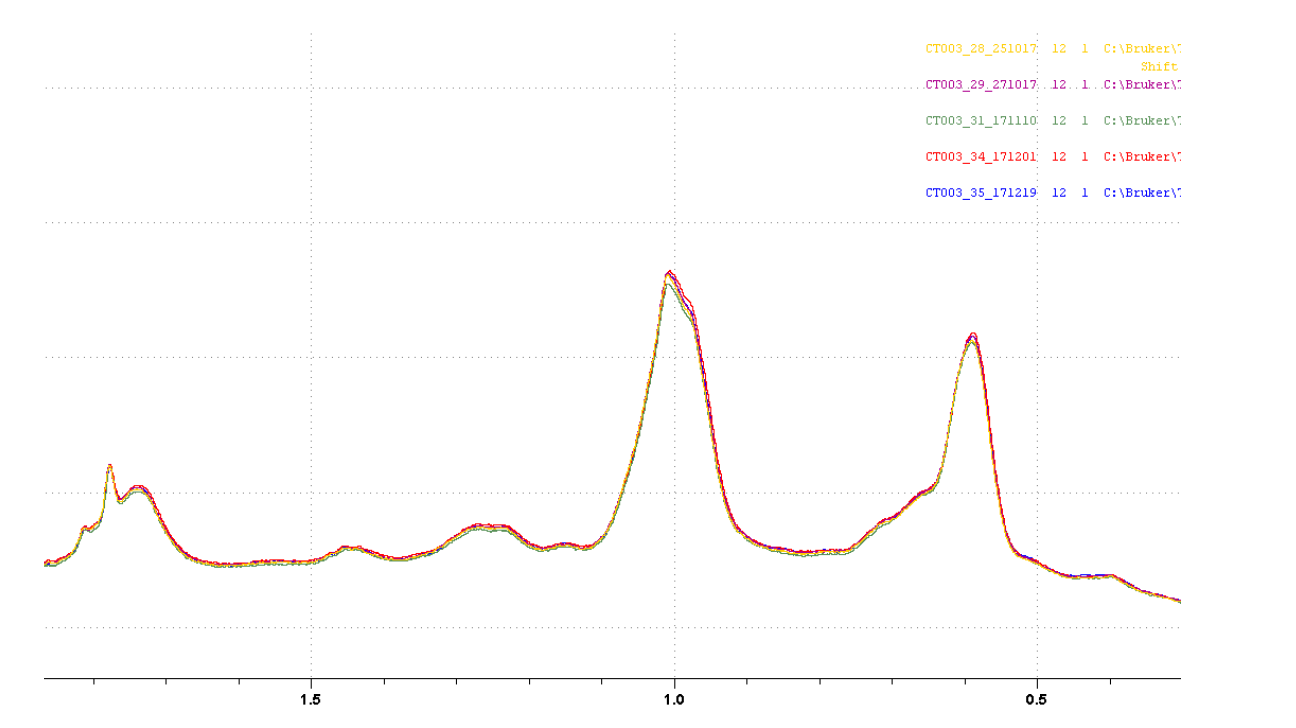

Supplement: S1 Fig — (TIF) [file pone.0341192.s001.tif]

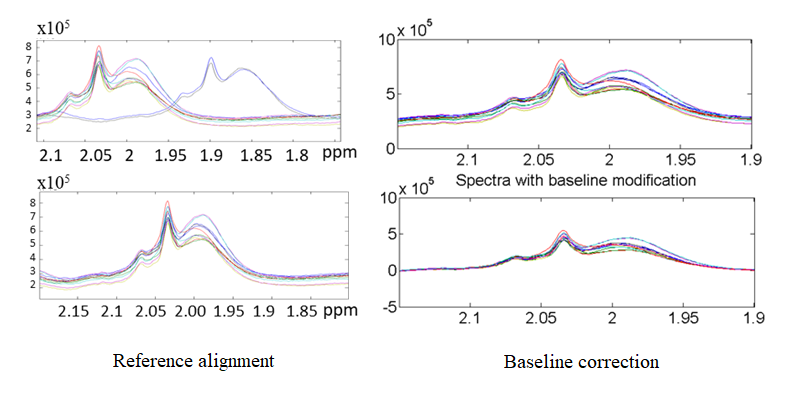

Supplement: S2 Fig — Automated spectral processing is integrated into the Liposcale® software, ensuring identical conditions for all samples. Referencing is performed using the glycoprotein region (2.15–1.90 ppm), aligning the main peak at 2.034 ppm. (TIF) [file pone.0341192.s002.tif]
