# Supplementary material for: Assessment of physical status and analysis of lipidomic and metabolomic alterations in patients with Post-COVID-19 condition
Source: PLoS One. 2026 Mar 3;21(3):e0341192. doi: 10.1371/journal.pone.0341192 (PMC12956072; doi:10.1371/journal.pone.0341192)
Supplement: S1 Table — The variables are represented by the median and the interquartile range. The comparison between the different categories was performed using the Kruskal-Wallis test. (DOCX) [file pone.0341192.s003.docx]

**S1 Table.** **Results of the lipoprotein analysis in the three study groups (control, COVID, and post-COVID) using 1H-NMR.** The variables are represented by the median and the interquartile range. The comparison between the different categories was performed using the Kruskal-Wallis test.

|  | **Control** | **COVID** | **post-COVID** | **p-value** |
| --- | --- | --- | --- | --- |
|  | *n=13* | *n=13* | *n=13* |  |
| **VLDL-Cholesterol (mg/dL)** | 9.92 [8.61-14.0] | 23.7 [18.7-31.5] | 12.0 [10.5-14.2] | <0.001 |
| **IDL-Cholesterol (mg/dL)** | 8.08 [6.14-10.1] | 19.9 [14.0-26.4] | 8.33 [6.88-9.70] | <0.001 |
| **LDL-Cholesterol (mg/dL)** | 118 [114-143] | 93.2 [82.1-106] | 116 [104-147] | 0.001 |
| **HDL-Cholesterol (mg/dL)** | 68.7 [66.3-71.4] | 42.2 [33.7-43.4] | 54.0 [50.4-56.5] | <0.001 |
| **VLDL-Triglycerides (mg/dL)** | 47.7 [35.0-70.1] | 78.6 [70.6-121] | 59.8 [48.2-69.1] | 0.003 |
| **IDL-Triglycerides (mg/dL)** | 9.27 [7.93-10.1] | 18.1 [14.1-23.9] | 9.84 [7.43-10.8] | <0.001 |
| **LDL-Triglycerides (mg/dL)** | 10.2 [9.23-13.6] | 20.5 [17.9-24.0] | 12.3 [9.77-15.3] | <0.001 |
| **HDL-Triglycerides (mg/dL)** | 12.6 [9.31-13.7] | 19.9 [15.2-22.5] | 12.2 [11.3-13.5] | <0.001 |
| **VLDL-P (nM)** | 33.1 [25.8-46.9] | 59.5 [53.7-90.3] | 42.2 [34.6-48.2] | 0.002 |
| **Large VLDL-P (nM)** | 0.87 [0.70-1.20] | 1.63 [1.32-1.81] | 1.23 [1.01-1.44] | 0.002 |
| **Medium VLDL-P (nM)** | 4.48 [3.48-5.73] | 7.23 [5.42-9.49] | 3.83 [3.62-5.13] | 0.009 |
| **Small VLDL-P (nM)** | 27.9 [22.2-38.7] | 50.9 [45.8-76.6] | 36.2 [30.0-42.8] | 0.001 |
| **LDL-P (nM)** | 1120 [1080-1366] | 1024 [892-1136] | 1190 [1041-1480] | 0.058 |
| **Large LDL-P (nM)** | 200 [184-231] | 166 [133-174] | 191 [162-202] | 0.003 |
| **Medium LDL-P (nM)** | 341 [296-461] | 305 [232-354] | 284 [268-456] | 0.313 |
| **Small LDL-P (nM)** | 611 [599-684] | 565 [415-635] | 728 [608-775] | 0.015 |
| **HDL-P (μM)** | 33.3 [30.2-35.6] | 21.2 [17.9-22.3] | 26.2 [24.0-27.1] | <0.001 |
| **Large HDL-P (μM)** | 0.29 [0.27-0.32] | 0.30 [0.26-0.33] | 0.29 [0.27-0.34] | 0.738 |
| **Medium HDL-P (μM)** | 11.2 [10.9-11.8] | 11.4 [10.8-12.0] | 10.4 [9.00-11.0] | 0.113 |
| **Small HDL-P (μM)** | 21.4 [17.9-23.6] | 9.10 [5.89-12.3] | 14.7 [14.0-15.7] | <0.001 |
| **VLDL-Diameter (nm)** | 42.4 [42.2-42.5] | 42.2 [41.9-42.2] | 42.1 [42.0-42.3] | 0.020 |
| **LDL-Diameter (nm)** | 21.3 [21.1-21.3] | 21.1 [21.0-21.3] | 21.0 [20.8-21.0] | 0.043 |
| **HDL-Diameter (nm)** | 8.25 [8.23-8.28] | 8.52 [8.41-8.68] | 8.33 [8.29-8.34] | <0.001 |
| **Non-HDL-P (nM)** | 1143 [1109-1403] | 1158 [968-1226] | 1272 [1075-1527] | 0.224 |
| **Total-P/HDL-P** | 1.04 [1.03-1.05] | 1.06 [1.05-1.07] | 1.05 [1.04-1.05] | 0.008 |
| **LDL-P/HDL-P** | 4.11 [3.99-4.41] | 3.34 [2.65-3.91] | 0.05 [0.04-0.05] | <0.001 |
| **Total Cholesterol (mg/dL)** | 208 [204-230] | 174 [162-196] | 189 [175-235] | 0.009 |
| **Total Triglycerides (mg/dL)** | 79.6 [65.7-103] | 143 [122-211] | 89.2 [83.0-109] | <0.001 |
| **VLDL-TG/VLDL-C** | 4.47 [4.11-5.00] | 3.82 [3.39-3.99] | 5.09 [4.60-5.44] | 0.006 |
| **IDL-TG/IDL-C** | 1.19 [1.15-1.28] | 0.98 [0.91-1.01] | 1.16 [1.08-1.28] | 0.001 |
| **LDL-TG/LDL-C** | 0.09 [0.08-0.10] | 0.25 [0.18-0.29] | 0.09 [0.08-0.11] | <0.001 |
| **HDL-TG/HDL-C** | 0.18 [0.15-0.19] | 0.47 [0.35-0.64] | 0.22 [0.19-0.24] | <0.001 |
