# Supplementary material for: Assessment of physical status and analysis of lipidomic and metabolomic alterations in patients with Post-COVID-19 condition
Source: PLoS One. 2026 Mar 3;21(3):e0341192. doi: 10.1371/journal.pone.0341192 (PMC12956072; doi:10.1371/journal.pone.0341192)
Supplement: S2 Table — The variables are represented by the median and the interquartile range. The comparison between the different categories was performed using the Kruskal-Wallis test. (DOCX) [file pone.0341192.s004.docx]

**S2 Table.** **Results of the glycoprotein analysis in the three study groups (control, COVID, and post-COVID) using 1H-NMR.** The variables are represented by the median and the interquartile range. The comparison between the different categories was performed using the Kruskal-Wallis test.

|  | **Control** | **COVID** | **post-COVID** | **p-value** |
| --- | --- | --- | --- | --- |
|  | *n=13* | *n=13* | *n=13* |  |
| **Glyc-B (μM)** | 297 [275-313] | 510 [445-528] | 331 [281-357] | <0.001 |
| **Glyc-F (μM)** | 188 [167-196] | 205 [182-257] | 208 [196-231] | 0.300 |
| **Glyc-A (μM)** | 592 [526-672] | 982 [868-1183] | 627 [612-645] | <0.001 |
| **H/W Glyc-B** | 3.73 [3.46-3.94] | 6.41 [5.59-6.64] | 4.16 [3.54-4.48] | <0.001 |
| **H/W Glyc-A** | 14.6 [13.3-15.9] | 24.1 [22.1-29.7] | 15.3 [13.4-17.3] | <0.001 |
