# Supplementary material for: Assessment of physical status and analysis of lipidomic and metabolomic alterations in patients with Post-COVID-19 condition
Source: PLoS One. 2026 Mar 3;21(3):e0341192. doi: 10.1371/journal.pone.0341192 (PMC12956072; doi:10.1371/journal.pone.0341192)
Supplement: S3 Table — The variables are represented by the median and the interquartile range. The comparison between the different categories was performed using the Kruskal-Wallis test. (DOCX) [file pone.0341192.s005.docx]

**S3 Table.** **Results of the lipid analysis in the three study groups (control, COVID, and post-COVID) using 1H-NMR.** The variables are represented by the median and the interquartile range. The comparison between the different categories was performed using the Kruskal-Wallis test.

|  | **Control** | **COVID** | **post-COVID** | **p-value** |
| --- | --- | --- | --- | --- |
|  | *n=13* | *n=13* | *n=13* |  |
| **Esterified cholesterol (mM)** | 2.56 [2.39-2.93] | 1.74 [1.42-2.10] | 2.66 [2.12-2.92] | 0.002 |
| **Free cholesterol (mM)** | 2.40 [2.2-2.44] | 2.29 [2.02-2.45] | 2.02 [1.83-2.52] | 0.492 |
| **Triglycerides (mM)** | 1.23 [0.79-1.58] | 1.54 [1.20-2.64] | 0.80 [0.48-0.97] | 0.022 |
| **Glycerophospholipids (mM)** | 3.69 [3.11-4.23] | 3.47 [3.14-3.66] | 3.42 [2.78-3.46] | 0.103 |
| **Phosphatidylcholine (mM)** | 2.80 [2.37-3.18] | 2.75 [2.52-2.98] | 2.71 [2.35-2.84] | 0.639 |
| **Sphingomyelin (mM)** | 1.00 [0.94-1.02] | 0.92 [0.80-1.00] | 0.81 [0.78-0.89] | 0.023 |
| **Lysophosphatidylcholine (mM)** | 0.88 [0.79-0.92] | 0.58 [0.55-0.61] | 0.76 [0.71-0.84] | <0.001 |
| **PLA** | . [.] | . [.] | 0.16 [0.11-0.23] | . |
| **PUFA (mM)** | 13.7 [13.5-14.4] | 13.8 [12.4-16.3] | 12.8 [11.0-14.5] | 0.415 |
| **Linoleic (mM)** | 4.01 [3.63-4.89] | 4.45 [3.60-5.21] | 4.14 [3.69-4.79] | 0.948 |
| **SFA (mM)** | 8.31 [5.39-11.0] | 10.7 [8.69-12.0] | 7.92 [7.31-8.64] | 0.019 |
| **w6 & w7 fatty acids (mM)** | 5.84 [5.07-6.93] | 5.83 [4.85-6.70] | 5.69 [4.52-5.87] | 0.351 |
| **w9 fatty acids (mM)** | 4.65 [2.10-7.65] | 3.81 [2.41-6.04] | 2.79 [1.86-3.40] | 0.151 |
| **w3 fatty acids (mM)** | 0.19 [0.17-0.24] | 0.19 [0.18-0.25] | 0.24 [0.18-0.26] | 0.418 |
| **DHA(mM)** | 0.10 [0.07-0.13] | 0.12 [0.07-0.13] | 0.11 [0.07-0.14] | 0.997 |
| **AA + EPA (mM)** | 1.24 [1.10-1.80] | 2.30 [2.06-2.56] | 1.12 [1.05-1.26] | <0.001 |
| **PUFA/SFA** | 1.65 [1.31-2.20] | 1.36 [1.15-1.46] | 1.66 [1.51-1.70] | 0.058 |
| **w6 & w7/w3** | 32.4 [25.3-37.1] | 25.6 [20.7-38.6] | 22.4 [18.5-32.5] | 0.267 |
