# Supplementary material for: Assessment of physical status and analysis of lipidomic and metabolomic alterations in patients with Post-COVID-19 condition
Source: PLoS One. 2026 Mar 3;21(3):e0341192. doi: 10.1371/journal.pone.0341192 (PMC12956072; doi:10.1371/journal.pone.0341192)
Supplement: S4 Table — The variables are represented by the median and the interquartile range. The comparison between the different categories was performed using the Kruskal-Wallis test. (DOCX) [file pone.0341192.s006.docx]

**S4 Table.** **Results of the low molecular weight metabolite (LMWM) analysis in the three study groups (control, COVID, and post-COVID) using 1H-NMR.** The variables are represented by the median and the interquartile range. The comparison between the different categories was performed using the Kruskal-Wallis test.

|  | **Control** | **COVID** | **post-COVID** | **p-value** |
| --- | --- | --- | --- | --- |
|  | *n=13* | *n=13* | *n=13* |  |
| **3-Hydroxybutyrate (μM)** | 26.5 [7.89-58.4] | 166 [107-301] | 11.0 [5.40-20.0] | <0.001 |
| **Acetone (μM)** | 7.55 [4.26-14.1] | 51.3 [19.1-114] | 8.62 [6.96-9.55] | <0.001 |
| **Alanine (μM)** | 260 [231-341] | 256 [229-367] | 298 [255-342] | 0.676 |
| **Creatinine (μM)** | 46.3 [41.9-48.8] | 31.7 [23.6-63.9] | 40.3 [32.4-46.2] | 0.547 |
| **Creatine (μM)** | 39.8 [35.1-54.5] | 50.7 [29.1-72.1] | 42.0 [21.7-43.9] | 0.146 |
| **Glucose (μM)** | 4205 [3733-4515] | 4339 [3950-6140] | 3054 [2732-3588] | 0.005 |
| **Glutamate (μM)** | 137 [81.4-227] | 151 [122-205] | 93.8 [79.4-108] | 0.007 |
| **Glutamine (μM)** | 265 [173-281] | 369 [328-394] | 346 [299-391] | 0.001 |
| **Glycerol (μM)** | . [.] | 211 [168-246] | 125 [86.3-141] | 0.001 |
| **Glycine (μM)** | 172 [127-224] | 269 [233-284] | 251 [234-266] | 0.006 |
| **Lactate (μM)** | 237 [151-350] | 1007 [881-1222] | 390 [313-442] | <0.001 |
| **Threonine (μM)** | . [.] | 257 [200-337] | 197 [173-248] | 0.053 |
| **Tyrosine (μM)** | 32.1 [28.7-34.2] | 42.5 [41.2-52.3] | 37.1 [28.4-41.1] | <0.001 |
| **Valine (μM)** | 146 [140-164] | 217 [184-254] | 159 [138-172] | <0.001 |
| **Isoleucine (μM)** | 17.7 [17.1-23.2] | 57.0 [44.7-91.4] | 39.9 [29.4-42.3] | <0.001 |
| **Leucine (μM)** | 63.2 [61.9-69.5] | 145 [111-154] | 76.2 [73.1-84.4] | <0.001 |
| **Glutamine/Glutamate** | 1.94 [0.73-3.85] | 2.40 [1.61-3.27] | 3.49 [3.15-4.56] | 0.020 |
